# Patients’ perception of the pharmacovigilance system: A pre-diagnostic and post-interventional cross-sectional survey

**DOI:** 10.3389/fphar.2022.936124

**Published:** 2022-11-17

**Authors:** Atta Ur Rehman, Samina Naeem Khalid, Rubeena Zakar, Ume Hani, Muhammad Zakria Zakar, Florian Fischer

**Affiliations:** ^1^ Health Services Academy, Quaid e Azam University, Islamabad, Pakistan; ^2^ Department of Public Health, Institute of Social and Cultural Studies, University of the Punjab, Lahore, Pakistan; ^3^ Holy Family Hospital, Rawalpindi Medical University, Rawalpindi, Pakistan; ^4^ Vice Chancellor Office, University of Poonch, Rawalakot, Pakistan; ^5^ Institute of Public Health, Charité—Universitätsmedizin Berlin, Berlin, Germany

**Keywords:** patients, perception, pharmacovigilance, health promotion, intervention

## Abstract

**Background and objective:** The risk of adverse reactions necessitated the pharmacovigilance system for patient safety. A literature search documented better health literacy of patients through intervention. This investigation aims to assess the perception and the effect of an intervention on patients regarding adverse reactions caused by drugs.

**Methods:** A pre-diagnostic and post-interventional cross-sectional investigation was designed with a sample size of 423 patients in hospitals of Islamabad. The proportion of patients was selected based on a stratified probability technique. A prevalidated tool was used to collect the response twice through a health promotion brochure with counseling, which was applied as an intervention.

**Results:** The outcome of the investigation revealed that the prerequisite of the pharmacovigilance center in the hospital among respondents was improved significantly by 41.2% after intervention. Knowledge, communication, and practice were significantly different with respect to gender. There was a moderate Pearson correlation between diagnostic and interventional responses of patient’s knowledge of adverse reactions by drugs (*r* = 0.66, *p* < 0.01) and patient’s communication in pharmacovigilance (*r* = 0.62, *p* < 0.01) and a strong correlation between diagnostic and interventional responses of patient’s practice in the pharmacovigilance system (*r* = 0.72, *p* < 0.01).

**Conclusion:** The finding of the investigation provided evidence that patient awareness was significantly improved by the health promotion model. Patient participation in the reporting of adverse reactions of drugs will complement the hospital staff reporting. These reports will construct an authentic, cross-checked database for rational drug safety practices in Pakistan.

## Introduction

Adverse reactions by drugs are significant healthcare threats to public health worldwide (Karimian et al., 2018). The adverse complications are escalating in patients due to disease co-morbidities that cause a forever-increasing demand for drugs (Chen et al., 2019). Drug-related complications were due to genetic variation, substandard medicine, under or overconsumption of prescribed dosage, irrational medicine usage, environmental conditions, lack of patient counseling, and non-adherence by patients (Belayneh et al., 2018). Adverse reactions by the same medication may differ between individuals and situations (Roden et al., 2011). Risk of adverse reactions necessitated the pharmacovigilance system for patients’ safety. Adverse reaction by drugs was one of the major causes of deaths associated with new hospitalizations worldwide (Giardina et al., 2018). Patients’ health care costs may be increased due to hospitalization for anti-dote therapy. Adverse reactions by drugs are indeed a financial burden to the patients, hospital administration, and the government (Sultana et al., 2018). The heavy cost of drug adverse responses may be envisaged that the patient’s belief is lost in the healthcare delivery system (Inacio et al., 2019).

The World Health Organization has reported that adverse reactions are often a reaction by the drug that is noxious and undesirable and usually develop at normal doses in disease diagnosis, prophylactic treatment, drug therapy, or to modify physiological processes (WHO, 2002). Pharmacovigilance is defined by the World Health Organization as “the science and activities related to the detection, assessment, understanding, and prevention of adverse drug effects or any other possible drug-related problems” (WHO, 2002). An adverse event or experience is defined as ‘any untoward medical occurrence that may present during treatment with a medicine but which does not necessarily have a causal relationship with this treatment (WHO, 2002). Patients are an important part of pharmacovigilance since they suffer from adverse drug reactions. The importance of adverse reactions by drugs is undoubtedly evident, but the adverse reactions are generally not documented or considerably under-reported by healthcare stakeholders (Adisa et al., 2019).

The main limitations in reporting were insufficient awareness about pharmacovigilance, non-availability of reporting documents in hospitals, and lack of knowledge about online reporting systems in patients. The patient-accessible online facility for adverse drug reaction reporting was offered by VigiBase, Uppsala Monitoring Center, Sweden; Food and Drug Administration, United States of America; MedWatch Yellow Card Scheme by the United Kingdom; and the Drug Regulatory Authority of Pakistan (Weigmann, 2016; Hussain and Hassali, 2019). Lack of pharmacovigilance awareness was observed in patients, and educative intervention was proposed to enhance responsiveness in Nepal (Jha et al., 2014).

The adverse drug reaction monitoring system is progressive in developed nations with the existence of a pharmacovigilance system at the hospital, regional, and national levels. The successful pharmacovigilance program of the Netherlands noticed dissimilarities in several reports by healthcare staff and patients due to differences in opinions about the severity and outcomes of adverse drug reactions (De et al., 2008). Patient reporting may initially be voluntary in low-income countries, but it must be mandatory after some time for a viable pharmacovigilance system. All of the stakeholders’ involvement may identify risk factors in a limited time to prevent or minimize adverse reactions (Pal et al., 2013).

Pakistan is the 134th Uppsala Monitoring Center participant in Sweden to record the pharmacovigilance activities (Hussain and Hassali., 2019). Health policy based on the Pakistan constitution considers health as an essential right of all the people. The Pakistan’s healthcare system is built on the national health policy (Jooma and Sabatinelli., 2013). Punjab Cardiology Institute, Lahore, recorded casualties of more than a hundred cardiac patients in 2012 as a result of adverse drug reactions from contaminated Isotab. This incident endorsed patient contributions in the reporting of adverse drug reactions in its true perspective to ensure rational drug use in the country (Hussain and Hassali., 2019). The purpose of patient involvement is to increase patient safety as being the actual target of these reactions. The scarcity of research in Pakistan related to the patient’s perception of the pharmacovigilance system provided evidence for an investigation. Therefore, a research survey was planned to determine the perception and effects of the intervention on patients regarding adverse reactions caused by drugs in hospitals of Islamabad.

## Materials and methods

### Survey design and population

A pre-diagnostic and post-interventional cross-sectional investigation was designed. The current survey was carried out in all the public sector tertiary care hospitals in the capital city of Pakistan. The hospital administration and institutional research review boards of the Pakistan Institute of Medical Sciences, Capital Development Authority Hospital, Federal Government, Chak Shahzad Hospital, Federal Government Polyclinic Hospital, and Social Security Hospital permitted the survey. The majority of the population of Islamabad visited outpatient departments of these hospitals for the treatment of diseases. All the tertiary care private healthcare facilities refused to allow the investigation in their premises. The survey populace involved both genders visiting the general medicine and general surgery outpatient departments. All the patients who gave willingness according to the recruiting strategy were involved in the survey.

### Sampled population size and technique for sampling

The sampled population size was assumed to be at 50% awareness prevalence with a 5% allowable error and confidence interval limits of 95% due to the non-availability of any published investigation in the country. The addition of a 10% non-responsive population in the Z formula resulted in 423 survey participants. The survey was based on quantitative research, and therefore probability techniques for sampling were considered. Because the hospital had average monthly records of patients’ visits, patients from each hospital were calculated by stratified random sampling as presented in Table 1. Patients from each stratum were chosen by a systematic random sampling technique. The first patient on the survey day was randomly chosen by the Sobol software method from the visitor’s token area/register, and then the regular interval “k” that was calculated for each hospital was added until the sample size was completed. If the nominee refused, then the next patient was contacted in turn. The refusal rate was quite high. The response rate in patients was 58%, as 423 questionnaires were received back twice out of 726 questionnaires distributed. The number of patients calculated from each hospital is elaborated in Table 1.

**TABLE 1:** Sample size calculation of patients from each hospital.

Name of the hospital	Average monthly patient visitors’ general medicine	Sample	Average monthly patient visitors’ general surgery	Sample
Pakistan Institute of Medical Sciences, Sector G 8	1,908	104	1,292	70
Federal Government Polyclinic Hospital, Sector G 6	1,802	98	1,248	68
Capital Development Authority Hospital	722	39	228	12
Federal Government, Chak Shahzad Hospital	229	12	71	4
Social Security Hospital	188	10	112	6
Total population	4,849	263	2,951	160

### Instrument for collecting data

The survey was based on a pre-validated instrument used in Nepal adopted from the Malaysian research study on pharmacovigilance. (Alshakka et al., 2007; Palaian et al., 2010; Jha et al., 2014). The questionnaire used in this survey was divided into four sections: patients’ demographics, patients’ knowledge of adverse reactions by drugs (patient’s immediate action after suffering from a disease, guidance provided by a healthcare professional for safe drug usage, patient’s compliance with healthcare guidance, patient’s understanding about the adverse reaction by drugs, patient’s perception regarding the purpose of reporting adverse reactions by drugs, vulnerable population for developing adverse reactions by drugs, appropriate person in the healthcare team for reporting adverse reactions by drugs, do you have the knowledge of pharmacovigilance as the science of detecting adverse drug reactions, and knowledge of online forms for reporting drug reactions), patient’s communication in pharmacovigilance (discussion with the physician about the probability of adverse drug reactions before taking medication, discussion with the physician about dose frequency and timing of medicines, discussion with the physician about precautions and instructions related to prescription, show compliance to prescriber instructions, and did/will you review the drug brochure about the adverse reaction of the drugs before taking the medication?), and patient practice in the pharmacovigilance system (experience of adverse drug reactions during the lifetime, did you report adverse drug reaction to anyone, I will be reporting adverse drug reactions in future, the prescribing and dispensing times should be improved to prevent adverse drug reactions, have you noticed/remembered any adverse drug reactions reported in the media, is there a need of pharmacovigilance center in hospitals, reporting of adverse reactions by drugs is beneficial for the populace as it reduces re-occurrence, and adverse drug reactions are a serious concern for healthcare stakeholders in Pakistan). The tool was modified on recommendations of expert professionals according to the local pharmacovigilance needs, in consistent with the literature published (Mahmood et al., 2011). A five-person expert committee was established, with members who have experience working for the Pakistani Drug Regulatory Authority, hospital staff members, and public health professionals. They were given the prevalidated research instrument and the intervention leaflet and were encouraged to make additional alterations. They suggested adding items such as adverse drug reactions are a serious concern for healthcare stakeholders in Pakistan and knowledge of online forms for reporting adverse drug reactions in the questionnaire with verbal inquiry about awareness of drug regulatory authority online reporting forms during intervention and counseling about availability as well as the reporting mechanism during the intervention. The committee decided on a format for the length and development of each item and reviewed and revised each newly proposed item. Ten patients from each institution participated in the initial pilot testing, which was followed by another review by an expert panel. Face validity of the instrument was judged by committee experts, and construct validity was analyzed using Pearson correlation. According to the Statistical Package for Social Sciences, the amended instrument’s reliability coefficient Cronbach’s alpha value was 0.90. Finally, these pilot results were included in the results as the total sample collected from each hospital.

### Health promotion model

The educational intervention took an average of 30 min excluding filling the time of pre- and post-intervention questionnaires. The interventional activity was completed during the waiting time of the patients in the outpatient department of the hospitals. The educational brochure comprised basic information regarding pharmacovigilance activities, awareness regarding side effects and adverse reactions to drugs, the procedure for suspected adverse reaction reporting, healthcare personnel’s role in monitoring and treatment, self-reporting websites, and the importance of reporting drug adverse reactions. After 10 min of the distribution of information brochures, counseling activity was conducted by trained pharmacists related to the information brochure.

### Statistical analysis and variables

Entry and analysis of data were based on SPSS software. The Statistical Software Package for Social Sciences version 21 was used (Dembe et al., 2011). The descriptive statistical analysis involved frequency and percentage calculations. The inferential investigation of data was computed on continuous variables formulated by summing identical items in an eight-item subscale for the awareness of adverse drug reactions by drugs, an eight-item subscale for the practice of adverse drug reactions by drugs, and a five-item subscale was formulated for communication in pharmacovigilance among patients. The Shapiro–Wilk test was performed to test the normality of the distribution. The chi-squared test, Pearson correlation, and paired *t*-test were applied on data for inferential inference.

### Ethics approval

The Ethics Review Board of the Health Services Academy, Islamabad, Pakistan, and institutional review boards/hospital administrators permitted the survey. The survey participants were informed about the project, and consent was obtained in writing.

## Results

### Patient demographics

Patient demographic data comprised patient’s age, gender, educational level, place of residence, and monthly income in Pakistani rupees. The respondents were divided into three main categories based on their age, education, and monthly income. The majority of the sampled population was in younger age groups (38.3%), uneducated (38.1%), and urban population (71.2%). Nearly half of the survey participants were female (54.4%). The majority (37.8%) earned between 50,000 and 100,000 Pakistani rupees every month. The patient demographics are represented in Table 2.

**TABLE 2:** Patient demographics.

Patient demographic	Frequency (percentage)
Age of respondents	
18–40 years	162 (38.3%)
41–60 years	145 (34.3%)
61 years and above	116 (27.4%)
Gender of respondents	
Male	193 (45.6%)
Female	230 (54.4%)
Residence of respondents	
Urban	301 (71.2%)
Rural	122 (28.8%)
Educational level of respondents	
Uneducated	161 (38.1%)
Up to intermediate level of education	154 (36.4%)
Graduate and above	108 (25.5%)
Monthly income of respondents(in Pakistani rupees)	
Less than 50,000	146 (34.5%)
50,000–100,000	160 (37.8%)
More than 100,000	117 (27.7%)

### Patient’s knowledge of adverse reactions by drugs

The questionnaire subsection related to patient’s knowledge by adverse reactions by drugs revealed that 74.2% of patients immediately consult doctors when suffering from the disease before the intervention, despite the fact that 16.1% replied that they will do self-medication when suffering from the disease in the future after counseling sessions and brochure intervention. The outcomes of the guidance provided by healthcare professionals for safe drug usage presented negligible improvement from 64.1% of patients to 68.8%. Patients’ compliance with the healthcare guide by the healthcare professionals was fully followed by 51.8% initially and 61.5% of the respondents in the final response. A total of 35.2% respondents knew that adverse reactions by the drug were the harmful response, while 75.2% participants identified correctly in the interventional survey. The data about the patient’s knowledge of adverse reactions by drugs in the pre-post analysis are presented in Table 3.

**TABLE 3:** Patients’ knowledge of adverse reactions by drugs and patients’ communication in pharmacovigilance.

Characteristic	Diagnostic response	Interventional response
	n (%)	n (%)
Patients’ knowledge of adverse reactions by drugs		
Patients’ immediate action after suffering from a disease		
a. Immediately consult the doctor for a prescription	314 (74.2%)	355 (83.9%)
b. Practice self-medication	109 (25.8%)	68 (16.1%)
Guidance provided by a healthcare professional for safe drug usage		
a. Yes	271 (64.1%)	291 (68.8%)
b. No	152 (35.9%)	132 (31.2%)
Patients compliance with healthcare guidance		
a. Completely	215 (50.8%)	260 (61.5%)
b. Not entirely	107 (25.3%)	89 (21%)
c. Not followed	101 (23.9%)	74 (17.5%)
Patients’ understanding about adverse reactions by drugs		
a. Harmful response by a drug*	149 (35.2%)	318 (75.2%)
b. Routine side effect	67 (15.8%)	57 (13.5%)
c. Desired response	59 (13.9%)	48 (11.3%)
d. Do not know	148 (35%)	0
Patients’ perception regarding the purpose of reporting adverse reactions by drugs		
a. Drug safety improve by reporting	80 (18.9%)	270 (63.8%)
b. Reoccurrence will be prevented	150 (35.5%)	107 (25.3%)
c. Prerequisite in the hospital setting	62 (14.7%)	42 (9.9%)
d. Enable physicians for early diagnosis	131 (31%)	4 (0.9%)
Vulnerable population for developing adverse reactions by drugs		
a. Child populace	139 (32.9%)	112 (26.5%)
b. Adult population	49 (11.6%)	31 (7.3%)
c. Old age people	82 (19.4%)	53 (12.5%)
d. All of the above	153 (36.2%)	227 (53.7%)
Appropriate person in the healthcare team for reporting adverse reactions by drugs		
a. Physician	84 (19.9%)	145 (34.2%)
b. Pharmacist	42 (9.9%)	58 (13.7%)
c. Nurse	42 (9.9%)	57 (13.5%)
d. All of the above	107 (25.3%)	163 (38.6%)
e. Do not know	148 (35.0%)	0
Do you have the knowledge of pharmacovigilance as the science of detecting adverse drug reactions?		
a. Yes	121 (28.6%)	414 (97.9%)
b. No	302 (71.4%)	9 (2.1%)
Knowledge of online forms for reporting drug reactions		
a. Yes	242 (57.2%)	420 (99.3%)
b. No	181 (42.8%)	3 (0.7%)
**Patients’ communication in pharmacovigilance**		
Discussion with the physician about the probability of adverse drug reactions before taking medication		
a. Yes	204 (48.2%)	317 (74.9%)
b. No	219 (51.8%)	106 (25.1)
Discussion with the physician about dose frequency and timing of medicines		
a. Yes	252 (59.6%)	403 (95.3%)
b. No	171 (40.4%)	20 (4.7%)
Discussion with the physician about precautions and instructions related to prescription		
a. Yes	267 (63.1%)	359 (84.9%)
b. No	156 (36.9%)	64 (15.1%)
Show compliance with prescriber instructions		
a. Yes	315 (74.5%)	359 (84.9%)
b. No	108 (25.5%)	64 (15.1%)
Did/will you review the drug brochure about the adverse reaction of the drugs before taking the medication?		
a. Yes	219 (51.8%)	370 (87.5%)
b. No	204 (48.2.%)	53 (12.5%)

*Correct response.

### Patient’s communication in pharmacovigilance

A total of 48.2% of the sampled population discussed the probability of adverse reactions by drugs before taking medication in the pre-survey, while the response was increased to 74.9% in the post-survey. As regards discussion about dose frequency and timing of medicine, 59.6% of participants before the intervention intended to discuss while 95.3% aimed to discuss it with the prescriber in future conversation. Precautions/instructions related to prescription were conversed with the physician by 63.1% of patients before the counseling session, and 84.9% of the patients intended to converse it with the prescriber in the forthcoming discussion. The data related to communication in pharmacovigilance among patients are presented in Table 3.

### Patient practice in the pharmacovigilance system

A total of 45.9% of the participants experienced adverse reactions by drugs during their lifetime, but the reporting rate was only 30.2%. The attitude toward reporting was modified by pharmacist counseling sessions and health brochure intervention, and 99.3% of respondents showed the intention to report in the future. Media reports were recalled by 56.7% of the patients in the initial response and 60.8% in the final response. The prerequisite of the pharmacovigilance center in hospitals was improved significantly from 56.7% to 97.9%. The data about pharmacovigilance practice among patients are described in Table 4.

**TABLE 4:** Patients’ practice in the pharmacovigilance system.

Characteristic	Diagnostic response	Interventional response
	n (%)	n (%)
Experience of adverse drug reactions during the lifetime		
a. Yes	194 (45.9%)	194 (45.9%)
b. No	229 (54.1%)	229 (54.1%)
Did you report adverse drug reactions to anyone?		
a. Yes	127 (30.02%)	127 (30.02%)
b. No	296 (69.98%)	296 (69.98%)
I will be reporting adverse drug reactions in future		
a. Yes	242 (57.2%)	420 (99.3%)
b. No	181 (42.8%)	3 (0.7%)
Prescribing and dispensing time should be improved to prevent adverse drug reactions		
a. Yes	252 (59.5%)	332 (78.5%)
b. No	171 (40.5%)	91 (21.5%)
Have you noticed/remembered any adverse drug reactions reported in the media?		
a. Yes	240 (56.7%)	257 (60.8%)
b. No	183 (43.3%)	166 (39.2%)
Is there need of pharmacovigilance centers in hospitals?		
a. Yes	240 (56.7%)	414 (97.9%)
b. No	183 (43.3%)	9 (2.1%)
Reporting of adverse reactions by drugs is beneficial for the populace as it reduces re-occurrence		
a. Yes*	260 (61.5%)	374 (88.4%)
b. No	163 (38.5%)	49 (11.6%)
Adverse drug reactions are a serious concern for healthcare stakeholders in Pakistan		
a. Yes*	226 (53.4%)	418 (98.8%)
b. No	197 (46.6%)	5 (1.2%)

*Correct response.

The patient’s knowledge of adverse reactions by drugs, patient’s communication in pharmacovigilance, and patient’s practice in the pharmacovigilance system were correlated with the intervention response. The findings of the bivariate Pearson correlation coefficient showed that there is a strong correlation between diagnostic and intervention responses of patient’s practice in the pharmacovigilance system and a moderate correlation between diagnostic and intervention responses of patient’s knowledge of adverse reactions by drugs and diagnostic and intervention responses of patient’s communication in pharmacovigilance. The outcome of intervention on paired variables showed significant differences (*p* ≤ 0.05). The results of pre-diagnostic and post-interventional assessments using the paired *t*-test are displayed in Table 5.

**TABLE 5:** Pre-diagnostic and post-interventional assessments using the paired *t*-test.

Variable	Response	Mean	SD	Mean difference	Correlation r-value	*p*-value	t-value df (422)	*p*-value
Patient’s knowledge of adverse reactions by drugs	Diagnostic response	18.33	4.49	4.94	0.66	<0.01	30.03	<0.01
	Interventional response	13.39	2.71					
Patient’s communication in pharmacovigilance	Diagnostic response	6.97	1.72	1.25	0.62	<0.01	18.90	<0.01
	Interventional response	5.72	0.89					
Patient’s practice in the pharmacovigilance system	Diagnostic response	11.80	2.46	1.80	0.72	<0.01	20.89	<0.01
	Interventional response	10.00	1.27					

The perception difference related to age, gender, and education of participants was computed by applying chi-squared statistics. The findings of the research investigation revealed that all the variables except the interventional response of communication in pharmacovigilance were significant for the age of the contributors. There were significant differences in gender among all variables. Communication in pharmacovigilance was only non-significant for education. The chi-squared statistics related to perception differences constructed on age, gender, and education are explained in Table 6.

**TABLE 6:** Perception differences based on age, gender, and education.

**Variable**	**Patient’s knowledge of adverse reactions by drugs**						
	**Response**	**Diagnostic response**	** $\chi^{2}$ **	***p*-value**	**Interventional response**	** $\chi^{2}$ **	***p*-value**
Age							
15–30 years	Don’t know	64.8%	63.61	<0.01*	61.6%	11.09	<0.01*
	Yes	35.2%			38.4%		
31–45 years	Don’t know	81.5%			43.2%		
	Yes	18.5%			56.8%		
46 and above years	Don’t know	33.9%			48.3%		
	Yes	66.1%			51.7%		
Gender							
Male	Don’t know	37.4%	95.28	<0.01*	32.3%	55.01	<0.01*
	Yes	62.6%			67.7%		
Female	Don’t know	83.6%			68.4%		
	Yes	16.4%			31.6%		
Education in years							
Uneducated	Don’t know	58.3%	52.53	<0.01*	56.4%	5.79	0.05
	Yes	41.7%			43.6%		
Matric and intermediate	Don’t know	81.9%			43.9%		
	Yes	18.1%			56.1%		
Graduate and above	Don’t know	38.1%			55,2%		
	Yes	61.9%			44.8%		
**Patient’s communication in pharmacovigilance**							
	**Response**	**Diagnostic response**	$\chi^{2}$	***p*-value**	**Interventional response**	$\chi^{2}$	***p*-value**
Age							
15–30 years	Don’t know	42.8%	7.47	0.02	51.6%	0.44	0.80
	Yes	57.2%			48.4%		
31–45 years	Don’t know	57.5%			49.3%		
	Yes	42.5%			50.7%		
46 and above years	Don’t know	44.9%			53.4%		
	Yes	55.1%			46.6%		
Gender							
Male	Don’t know	13.6%	180	<0.01*	30.8%	62.56	<0.01*
	Yes	86.4%			69.2%		
Female	Don’t know	79.1%			69.3%		
	Yes	20.9%			30.7%		
Education in years							
Uneducated	Don’t know	41.7%	5.17	0.07	49.1%	4.28	0.11
	Yes	58.3%			50.9%		
Matric and intermediate	Don’t know	54.2%			47.7%		
	Yes	45.8%			52.3%		
Graduate and above	Don’t know	50.5%			60%		
	Yes	49.5%			49%		
**Patient’s practice in the pharmacovigilance system**							
	**Response**	**Diagnostic response**	$\chi^{2}$	***p*-value**	**Interventional response**	$\chi^{2}$	***p*-value**
Age							
15–30 years	Don’t know	54.7%	77.79	<0.01*	80.05%	37.40	<0.01*
	Yes	45.3%			19.5%		
31–45 years	Don’t know	8.2%			47.3%		
	Yes	91.8%			52.7%		
46 and above years	Don’t know	45.8%			58.5%		
	Yes	54.2%			41.5%		
Gender							
Male	Don’t know	23.7%	24.92	<0.01*	58.6%	2.94	0.05
	Yes	76.3%			41.4%		
Female	Don’t know	47.1%			66.7%		
	Yes	52.9%			33.3%		
Education in years							
Uneducated	Don’t know	49.7%	78.89	<0.01*	71.2%	26.25	<0.01*
	Yes	50.3%			28.8%		
Matric and intermediate	Don’t know	9.0%			47.1%		
	Yes	91.0%			52.9%		
Graduate and above	Don’t know	55.2%			73.3%		
	Yes	44.8%			26.7%		

Note: * (significant at ≤ 0.05 *p*-value).

## Discussion

Patients’ perception of the disease and drugs plays a vital role in the successful therapy model in health management. Patients’ education involves counseling that is important for disease understanding and awareness of pharmacological and non-pharmacological approaches in the treatment. Compliance with therapy may be improved by effective active and passive counseling of the patients. Active counseling involves face-to-face conversation, while passive counseling involves the use of written information (Saood et al., 2020). The current investigation involved a mixed method of face-to-face counseling sessions with health brochure intervention. A total of 423 patients from the targeted outpatient departments were evaluated for the survey. The majority of the sampled population was women because of the high incidence of disease, also testified by Gove (1984). Self-ingestion of medicines without physician consultation was reported as one of the significant etiological cause of adverse drug reaction (Mahmood et al., 2011). The present research investigation reported a decrease in patient’s intention toward self-medication when they suffered from disease after the intervention.

The World Health Organization documented that the majority of the patients globally fail to take medicines correctly (World Health Organization, 2004). The poor drug adherence contributing factors are related to patients and physicians. The barrier in communicating with the physician and lack of communication in pharmacovigilance were the most important physician-contributing factors (Brown et al., 2011). Similar results were reported by the majority of the patients in this survey. The suboptimal level of health literacy in patients evoked poor compliance (Millar et al., 2016). The instructions of the prescriber were fully followed by less than 62% of the patients after intervention. Low health literacy of the patients may be linked with uneducated and less educated participants in the survey. The majority of the respondents were not able to recognize the concept of adverse reactions by drugs in the diagnostic survey. Perception regarding adverse drug reactions was also low in some areas of Nepal and Nigeria (Jha et al., 2014; Adisa et al., 2019). The understanding of adverse drug reactions was improved in two-thirds of the participants after health communication. Almost half of the counseled respondents correctly identified vulnerable populations to develop adverse drug reactions. The literature search also nullified the concept that the children were most susceptible to adverse reactions by drugs. Everyone may be endangered to adverse reactions by drugs, irrespective of age group, sex, race, and other factors (Mahmood et al., 2011; Inacio et al., 2017). The familiarity of the pharmacovigilance concept was less in 1/3rd of the sampled population in the diagnostic survey also reported in fifty nations’ metanalysis reports on adverse drug reactions (Margraff et al., 2014). The intervention created awareness in more than 95% of the participants. The knowledge of patients regarding an appropriate person in the healthcare team is a prerequisite for reporting. Healthcare team members of all specialties were involved in signal detection in pharmacovigilance systems globally. The majority of the patients did not appropriately recognize the responsible person for reporting adverse drug reactions in the diagnostic survey, but after the intervention, they were able to identify the health personnel’s involvement in adverse drug reactions. Patients’ reporting had generated positive outcomes in the previous literature and is a prerequisite of the day for patient’s safety (Mahmood et al., 2011).

The Eric’s report declared that drug safety data need to be transmitted effectively for educating healthcare stakeholders, so that the risk–benefit data of medicines may be interpreted timely, and the exchange of such data at the international and national levels should be recommended (Hugman, 2006). The health conversation of patients with prescribers related to the effects of adverse drug reactions was aimed to be less than 75% in the future response. The Patient–physician communication of dose and timing is important because type A reactions are dose-dependent (Coleman and Pontefract, 2016). Dose and timing of medication conversation with the physician were improved significantly to 95.3% in the final response. The lack of patient compliance with therapy resulted in resistance to treatment, therapy failure, deaths, prolonged hospital duration, and increased expenditure on healthcare. Chronic medication adherence was detected 50% in patients (DiMatteo et al., 2002). The patients’ compliance with prescriber’s instructions was increased to 10.4% in the final response. The drug leaflet guide is a source for providing relevant information to consumers (Adepu and Swammy, 2012). The percentage of future drug literature reviewers increased to 35.8% after intervention.

A substantial number of participants (45.9%) declared that they experienced adverse reactions by drugs, and only 30.2% documented that to health personnel. Medical professionals’ poor knowledge in signal detection and rare practice of reporting are major constraints in a viable pharmacovigilance system in the countries (Fernandopulle and Weerasuriya, 2003; AlShammari and Almoslem, 2018). The majority of patients intended in both surveys for future reporting of adverse reactions by drugs. Medical professionals’ underreporting in developing nations will be supplemented by an autonomous patient pharmacovigilance reporting practice. The consumer reporting of adverse reactions may be a beneficial project for safety assurance. The majority of the patients were able to recall media reports; therefore, the potential of the media should be utilized in Pakistan for the dissemination of pharmacovigilance reports (Van Hunsel et al., 2009). Patients believed that physician’s prescribing time and pharmacist dispensing time should be improved for a better understanding about drugs. Mostly, patients proposed a hospital-based pharmacovigilance system in the country for effective health communication among stakeholders for patients’ safety (Saqib et al., 2019) The personalized drug model proposed by Wertheimer may guide for efficient, suitable, economical, and safe drug usage globally (Wertheimer, 2017).

The findings of this survey revealed that diagnostic and interventional response variables were moderately and positively correlated in patient’s knowledge and patient’s communication and strongly and positively correlated in patient’s practice in the pharmacovigilance system. There was a significant average difference between diagnostic and interventional responses in all the three testing components. The mean values were higher for diagnostic responses, and the differences were statistically significant. The average difference in patients’ knowledge was 4.94, whereas it was 1.25 in patients’ communication and 1.80 in patients’ practice. The majority of the population started choosing correct responses after intervention. There was little variation in average and standard variation in comparison to the diagnostic response.

## Limitations

There are some limitations to this survey: first, only public sector hospitals in the federal capital permitted the study; therefore, the information may not represent the patients from the private hospitals. Second, due to time limitation, only general surgery and general medicine departments were included. However, this pioneer survey of the fifth most populated nation may provide a basis for further investigations related to patients. The research was carried out in Pakistan’s federal capital, and the results will only be cautiously extrapolated to the nation as a whole. There is a need for further research to investigate the predictors, promoters, and barriers in adverse reaction reporting among patients in Pakistan.

## Conclusion

The results of the pioneer survey concluded that health literacy improved significantly in the interventional survey, but the baseline results indicated a low awareness level of pharmacovigilance among patients in the federal capital of Pakistan. The survey revealed that the majority of the participants were interested in physician consultation for drug use; some were willing to report adverse drug reactions in the future and demanded the establishment of a pharmacovigilance system at the hospital level. Patients’ participation in the reporting of adverse reactions of drugs will complement the hospital staff reporting. These reports will construct an authentic, cross-checked database for rational drug safety practices in Pakistan.

## Data Availability

The raw data supporting the conclusion of this article will be made available by the authors, without undue reservation.
